# Supplementary material for: A contextual fear conditioning paradigm in head-fixed mice exploring virtual reality
Source: bioRxiv. 2025 Apr 13:2024.11.26.625482. Originally published 2024 Nov 27. Preprint. [Version 2] doi: 10.1101/2024.11.26.625482 (PMC11623582; doi:10.1101/2024.11.26.625482)
Supplement: Supplement 1 [file media-1.pdf]

Supplementary videos can be downloaded from the following link:
https://uchicago.box.com/s/t1nkkadmv1bt6wa8ab3y32b5vel7x9x7
