# Supplementary material for: A contextual fear conditioning paradigm in head-fixed mice exploring virtual reality
Source: bioRxiv. 2025 Apr 13:2024.11.26.625482. Originally published 2024 Nov 27. Preprint. [Version 2] doi: 10.1101/2024.11.26.625482 (PMC11623582; doi:10.1101/2024.11.26.625482)
Supplement: 2 [file NIHPP2024.11.26.625482v2-supplement-2.pdf]

bioRxiv preprint doi: https://doi.org/10.1101/2024.11.26.625482; this version posted April 13, 2025. The copyright holder for this preprint (which was not certified by peer review) is the author/funder, who has granted bioRxiv a license to display the preprint in perpetuity. It is made available under aCC-BY 4.0 International license.

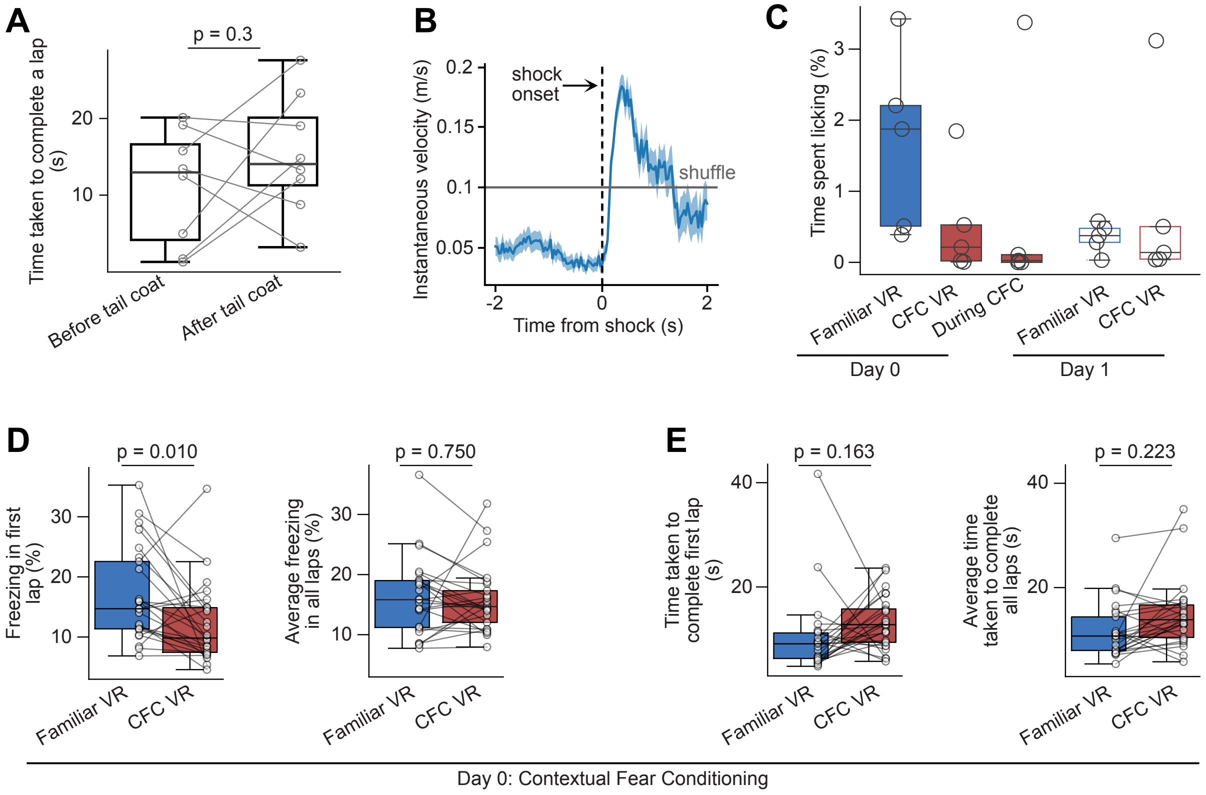

Supplementary Figure 1. Running and licking behaviors in Paradigm 1. (A) Box plot of average time taken to complete a lap before and after the addition of a tail-coat recorded in a randomly chosen subset of mice (circles, n=8). **(B)** Instantaneous velocity increased immediately after shock onset. This increased running speed also served as a reliable measure to confirm that mice received the tail shock. The grey line indicates the average velocity calculated by shuffling the instantaneous velocity 100 times. **(C)** Licking behavior doesn't stop immediately in the familiar VR without water, as mice previously received water rewards in the familiar VR. However, it was low in the other sessions and across environments. There was no significant difference between the Familiar and CFC VR on Day 1. Licking behavior was collected only in a subset of mice (circle, n=5). **(D-E)** The amount of freezing **(D)** and time taken to complete a lap **(E)** were not significantly different between the Familiar and CFC VR before fear conditioning (n=25). While not significantly different, there was a trend towards longer time taken in the first lap of the CFC VR **(E)**, which we have observed before when mice enter novel environments from familiar ones (Dong et al., 2021). The significantly lesser freezing during this first lap **(D)** suggests that animals were exploring slowly rather than remaining immobile. The boxplots (in A, C-E) range from the first quartile (25th percentile) to the third quartile (75th percentile), and the box shows the interquartile range (IQR). The line across the box represents the median (50th percentile). The whiskers extend to 1.5*IQR on either side of the box, and anything above this range is defined as an outlier. P-values were calculated using a paired *t-test.*

bioRxiv preprint doi: https://doi.org/10.1101/2024.11.26.625482; this version posted April 13, 2025. The copyright holder for this preprint (which was not certified by peer review) is the author/funder, who has granted bioRxiv a license to display the preprint in perpetuity. It is made available under aCC-BY 4.0 International license.

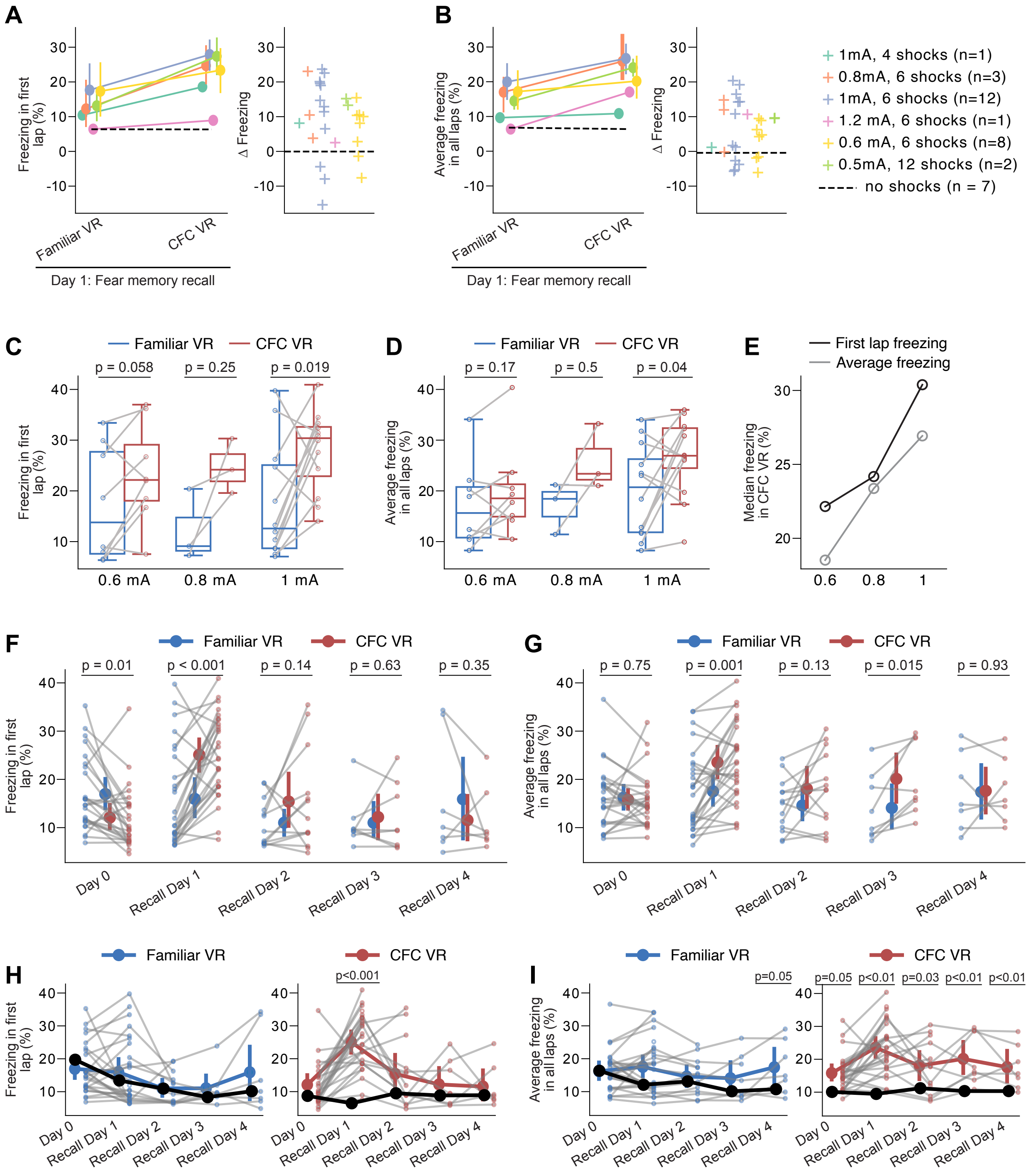

Supplementary Figure 2. Change in freezing with shock amplitude, number of shocks, and across recall days in Paradigm 1. (A-B) The left panel shows freezing in the first lap **(A)** and across all laps **(B).** The right panel illustrates delta freezing, calculated as the difference in freezing between the CFC VR and the Familiar VR. The colors represent the shock parameters used. Number of animals used is indicated in the figure legend. The dashed line represents the average freezing percentage in a control paradigm, where mice experienced the same conditions without shocks (n = 7). In most cases, we observed an increase in freezing in the CFC VR compared to the Familiar VR (delta>0). **(C-D)** Freezing in the first lap **(C)** and across

bioRxiv preprint doi: https://doi.org/10.1101/2024.11.26.625482; this version posted April 13, 2025. The copyright holder for this preprint (which was not certified by peer review) is the author/funder, who has granted bioRxiv a license to display the preprint in perpetuity. It is made available under aCC-BY 4.0 International license.

all laps **(D)** by shock amplitude. In all cases, mice received six shocks that were 1 minute apart. Greater fear discrimination could be seen at higher shock amplitudes. **(E)** Median freezing in the CFC VR by shock amplitude for first lab freezing (black) and average freezing across laps (gray). **(F-G)** A subset of animals underwent multiple days of recall tests (n = 25 Day 0, n = 27 Recall Day 1, n = 13 Recall Day 2, n = 8 Recall Day 3, n = 7 Recall Day 4). VR environments were presented in a counterbalanced manner for 5 minutes each day. The increase in freezing in the CFC VR was highest on the first day, both in the first lap **(F)** and across all laps **(G).** P-values were calculated using a paired *t-test.* **(H-I)** Within-VR comparison of the same dataset. First lap freezing **(H)** and average freezing across all laps **(I),** separated by Familiar VR (left panels) and CFC VR (right panels), demonstrate fear extinction across days. The black line represents average freezing in control mice that received no shocks. P-values indicate significant differences between experimental and no-shock control groups (t-test). Fear extinction is quantified by the LME model in Tables 1 and 2, which shows the statistical comparison of freezing in recall days versus Day 0 (baseline) within each VR.

bioRxiv preprint doi: https://doi.org/10.1101/2024.11.26.625482; this version posted April 13, 2025. The copyright holder for this preprint (which was not certified by peer review) is the author/funder, who has granted bioRxiv a license to display the preprint in perpetuity. It is made available under aCC-BY 4.0 International license.

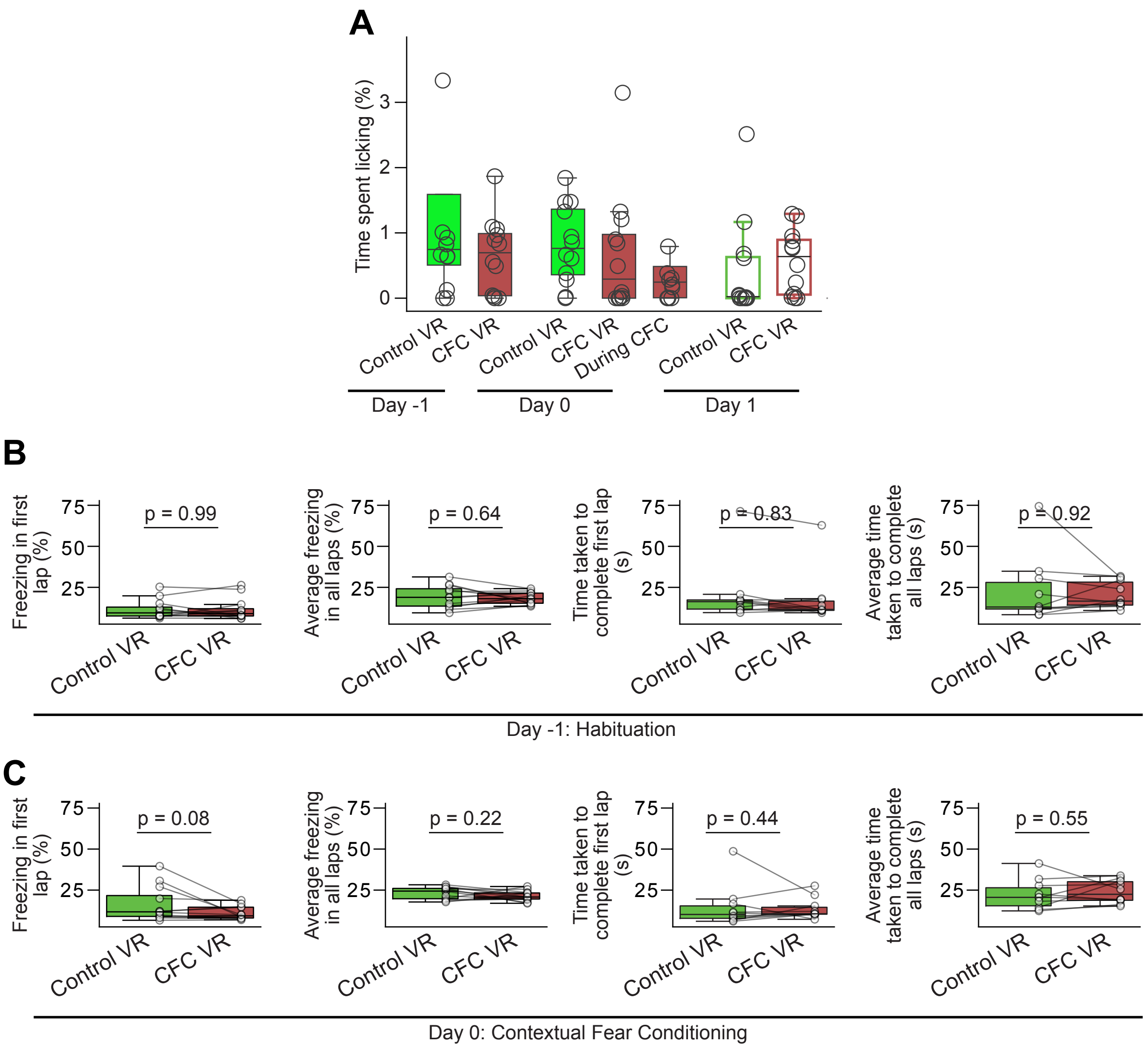

Supplementary Figure 3. Licking and running behavior before fear conditioning, in Paradigm 2. (A) Licking behavior remains low in all sessions, unlike Paradigm 1. It does not significantly differ between the Control vs CFC VR before and after CFC. **(B-C)** The amount of freezing and average running speed was not significantly different between the Control and CFC VR before fear conditioning, both on Day -1 **(B)** and Day 0 **(C)**. P-values were calculated using a *paired t-test.*

bioRxiv preprint doi: https://doi.org/10.1101/2024.11.26.625482; this version posted April 13, 2025. The copyright holder for this preprint (which was not certified by peer review) is the author/funder, who has granted bioRxiv a license to display the preprint in perpetuity. It is made available under aCC-BY 4.0 International license.

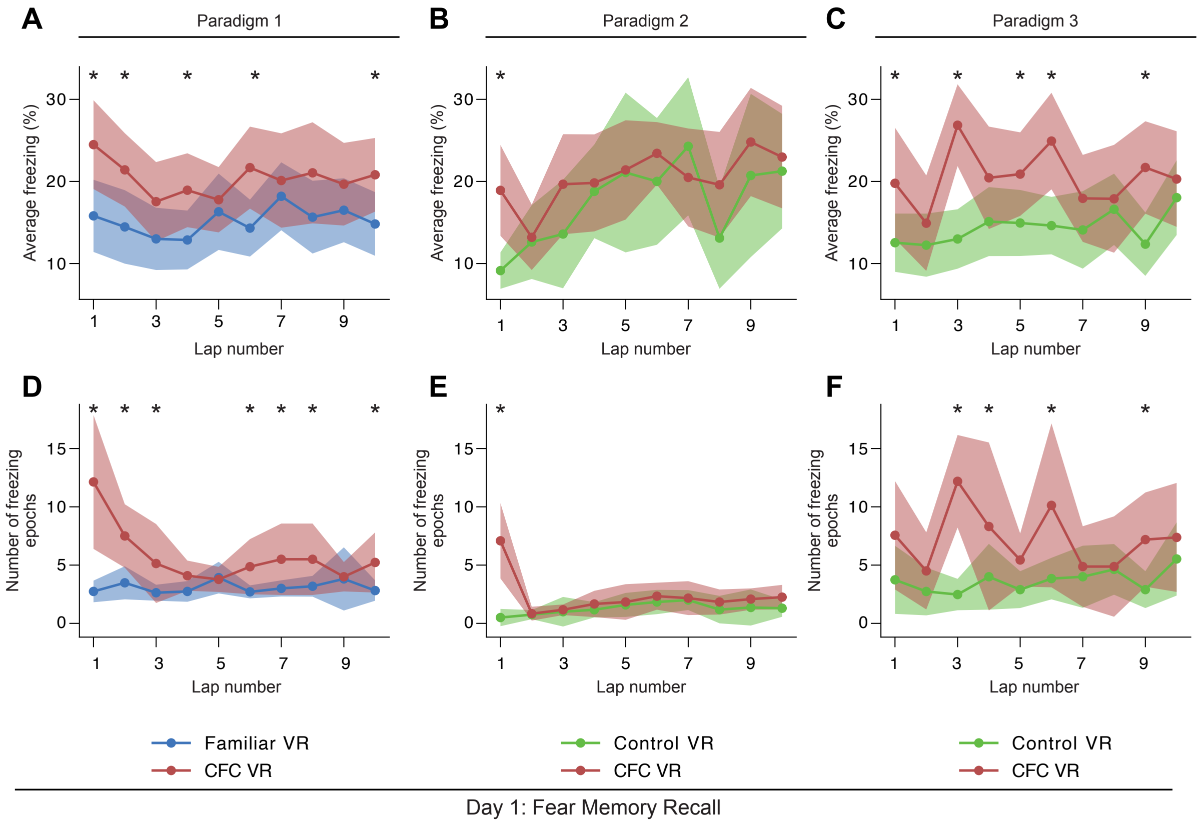

Supplementary Figure 4. Lap-wise freezing behavior in the three paradigms. (A-F) Average freezing across mice **(A-C)** and the number of freezing epochs (**D-F,** see Methods) in each lap across the two VRs on the first day of recall in **(A, D)** Paradigm 1, **(B, E)** Paradigm 2 and **(C, F)** Paradigm 3. Lines indicate mean, and shading indicates 95% confidence intervals. P-values between **(A, D)** Familiar VR and CFC VR and **(B-C, E-F)** Control VR and CFC VR were calculated using a *paired t-test.* * indicates p-values<0.01.

bioRxiv preprint doi: https://doi.org/10.1101/2024.11.26.625482; this version posted April 13, 2025. The copyright holder for this preprint (which was not certified by peer review) is the author/funder, who has granted bioRxiv a license to display the preprint in perpetuity. It is made available under aCC-BY 4.0 International license.

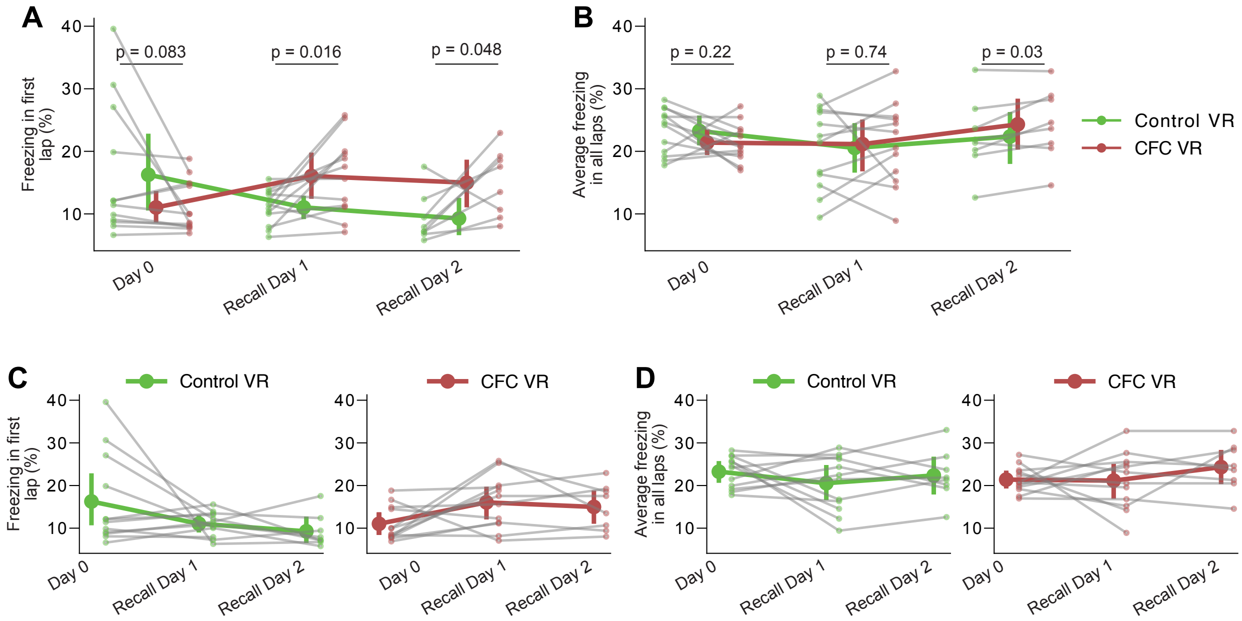

Supplementary Figure 5. Mice largely extinguished their fear within the first day of recall in Paradigm 2. (A-B) First lap freezing **(A)** and average freezing across all laps **(B)** across two days of recall in Paradigm 2. P-values were calculated using a paired t-test. Circles indicate data from individual mice (n = 12 for Recall Day 1 and n = 8 for Recall Day 2). P-values were calculated using a paired *t-test.* (**C-D**) Within-VR comparison of the same dataset. First lap freezing **(C)** and average freezing across all laps **(D),** separated by Familiar VR (left panels) and CFC VR (right panels), demonstrate fear extinction across days. Fear extinction is quantified by the LME model in Tables 3 and 4, which shows the statistical comparison of freezing in recall days versus Day 0 (baseline) within each VR.

bioRxiv preprint doi: https://doi.org/10.1101/2024.11.26.625482; this version posted April 13, 2025. The copyright holder for this preprint (which was not certified by peer review) is the author/funder, who has granted bioRxiv a license to display the preprint in perpetuity. It is made available under aCC-BY 4.0 International license.

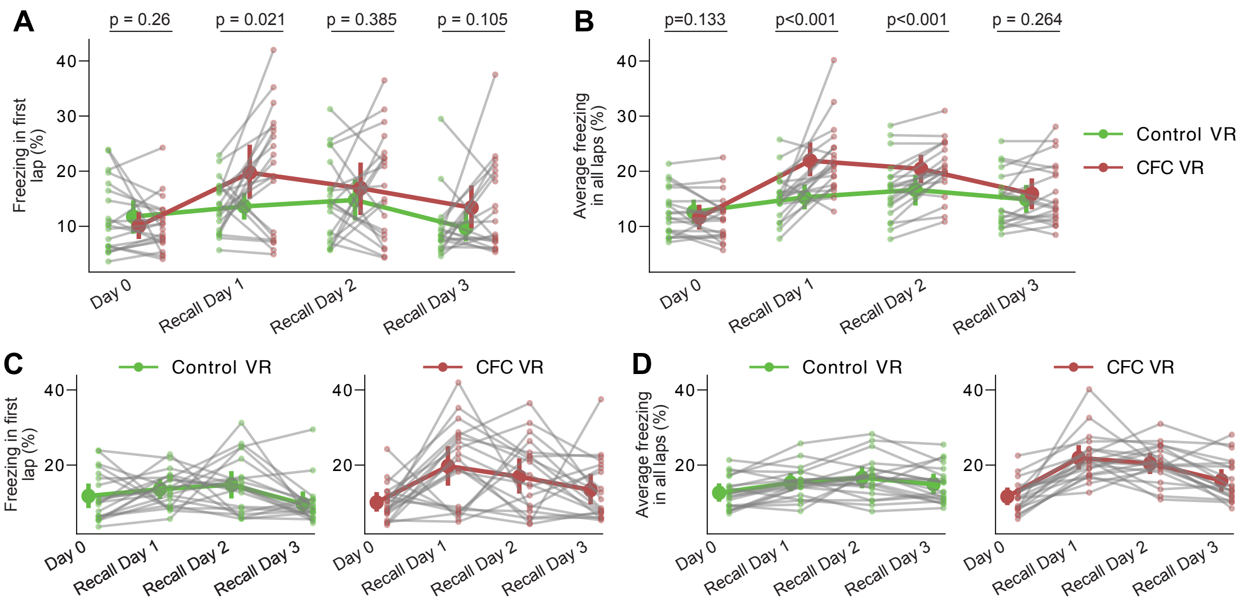

Supplementary Figure 6. Mice displayed better fear discrimination and a delay in extinction in Paradigm 3 compared to Paradigms 1 and 2 (A-B) Freezing in the first lap **(A)** and average freezing across all laps **(B)** in the Control VR vs CFC VR on three consecutive recall days in Paradigm 3 (n = 20). The tail-coat was kept on all recall days. P-values were calculated using a paired *t-test.* (**C-D**) Within-VR comparison of the same dataset. First lap freezing **(C)** and average freezing across all laps **(D),** separated by Familiar VR (left panels) and CFC VR (right panels), demonstrate fear extinction across days. Fear extinction is quantified by the LME model in Tables 5 and 6, which shows the statistical comparison of freezing in recall days versus Day 0 (baseline) within each VR.

bioRxiv preprint doi: https://doi.org/10.1101/2024.11.26.625482; this version posted April 13, 2025. The copyright holder for this preprint (which was not certified by peer review) is the author/funder, who has granted bioRxiv a license to display the preprint in perpetuity. It is made available under aCC-BY 4.0 International license.

Supplementary Video Legend

Supplementary Videos 1, 2 and 3: Example video of a head-fixed mouse navigating on a treadmill in VR. The examples show mouse behavior on Recall Day 1 in Paradigm 1 in: **1)** the Familiar VR and **2 and 3)** the CFC VR. Video 2 shows freezing behavior in CFC VR and Video 3 shows another mouse displaying more avoidance-like behaviors like slowing down and backward movements. Videos were recorded for a few seconds immediately after the mouse was transitioned to each VR environment. Videos were collected at a sampling rate of 30 frames/sec.
